# Confidence in dementia care and care approach toward dementia among the nursing staff in long-term care facilities in China: a cross-sectional survey

**DOI:** 10.3389/fpubh.2023.1182631

**Published:** 2023-08-17

**Authors:** Haiwen Chen, Jie Song, Nan Zhang, Na Li, Qianqian Jiang, Xiaohan Lu, Lin Liu, Yue Liu

**Affiliations:** ^1^School of Nursing, Shandong University of Traditional Chinese Medicine, Jinan, China; ^2^Jinan Social Welfare Institute, Jinan, China; ^3^Provincial Hospital Affiliated to Shandong First Medical University, Jinan, China

**Keywords:** dementia, self confidence, person-centered care, long-term care, nursing staff, cross-sectional study

## Abstract

**Background:**

Confidence and appropriate care approach toward dementia among nursing staff (nurses and care assistants) are crucial manifestations of competency to deal with the complexity of dementia care in long-term care facilities (LTCFs). The purpose of this study was to investigate the confidence in dementia care and care approach among nursing staff in LTCFs in mainland China.

**Methods:**

A cross-sectional study design was utilized in LTCFs in Shandong Province, mainland China. A convenient sample included of 317 nursing staff drawn from 15 LTCFs. Survey questions included (a) demographics, (b) dementia knowledge, (c) dementia care confidence, and (d) approach to care for people with dementia. Data were analyzed with descriptive statistics. Factors associated with confidence and care approach for people with dementia were examined using Pearson’s correlation and multivariate regression analyses.

**Results:**

Dementia care confidence was generally moderate. Factors affecting confidence to care for people with dementia included educational level, months of caring dementia patients, and dementia knowledge. Most nursing staff did not use a person-centered care approach which was significantly associated with their age, dementia-learning experience, and knowledge and confidence toward caring for people with dementia.

**Conclusion:**

A positive correlation was identified between confidence to care for people with dementia and nursing staff care approach. Clinical recommendations are provided to further develop education strategies tailored for nursing staff to meet the growing demand for dementia care services.

## 1. Introduction

As one of the main threats to the health and quality of life for older people worldwide, dementia is a syndrome characterized by a decline in memory or other cognitive functions that affect person’s ability to perform daily activities (1). Based on the World Health Organization’s report, about 55 million people worldwide suffered from dementia in 2021, and this number is expected to be over 139 million by 2050 (2). There are currently more than 9.5 million people with dementia in China, with this number expected to exceed 40 million by 2050 due to the rapid aging process, imposing heavy economic and care-giving burden on the public and health care system (3). In the Chinese culture, the Confucian practice of “respecting the old” is largely valued. Therefore, comprehensive dementia care of parents has long been provided by adult children at home (4). However, in the past several years, with the growth of population mobility and the changes of family structure in numerous Chinese families, adult children are unavailable to provide care for their parents with dementia (5). As a result, the long-term care facilities (LTCFs) have become the alternative, practical selection for people with dementia. Especially, in developed countries, approximately 80% of residents lived with dementia in LTCFs and this number is expected to increase as the population ages (6).

Although the number of people with dementia in LTCFs is increasing, few nursing staff have adequate knowledge, skills, and confidence in dementia care (7). In recent years, interest in assessing confidence to work with people with dementia has been growing rapidly. Staff in nursing home generally perceived people with dementia as “anxious, frightened, and having little control over their behavioral and psychological symptoms of dementia (BPSD)” (8). The complexity of caring for people with dementia may lead to a generally decreased confidence level among nursing staff in the LTCFs. Furthermore, confidence in dementia care could be influenced by the nursing staff lack of dementia knowledge (9). For example, a survey conducted in 261 nursing staff working in the public nursing and residential homes in Malta showed that their confidence in caring for people with dementia was low and could be improved by a 14 h training program on the knowledge about dementia management, care, and policy (10), while the similar results were observed among staff members from three National Health Service Institutions located in North-West England (11).

According to Holton’s model, confidence in acquired knowledge can successfully motivate learners to transfer the knowledge to intentions and actions (12). Based on this well-established model, nursing staff with higher level of knowledge and better confidence in dementia were more likely to adopt the person-centered dementia care approach compared to those with deficient knowledge and confidence (13). The person-centered dementia care approach rather than the reality-oriented approach was beneficial for care recipients (14). For example, a systematic review suggested that the reality-oriented dementia care approach had limited ability to relieve detachment, depressive symptoms, and agitated behavior in people with dementia in middle to late stage (15). Furthermore, the reality-oriented care approach emphasized constant correction of time, place, and character may aggravate the severity of dementia symptoms (16, 17). However, the person-centered approach could reduce neuropsychiatric symptoms, agitation, and depression as well as improve the quality of life for people with dementia, while relieving the pressure and burden of nursing staff (18, 19). Unfortunately, the person-centered approach has not been widely applied by nursing staff for people with dementia. For example, Lin et al. (20) conducted a cross-sectional survey of a total of 124 nurses from a hospital in southern Taiwan to evaluate their method of providing care for people with dementia. This study showed that 67.69% of the 124 nurses chose reality-oriented dementia care, whereas older and more experienced nurses tended to use a person-centered approach. Similarly, nursing staff who received basic dementia education were aware of person-centered dementia care and were concerned more about personality rather than custodial care (21).

In summary, increasing numbers of people with dementia require a qualified nursing workforce in LTCFs (22). Unfortunately, nursing staff confidence regarding working with people with dementia was low and the care approach did not promote positive outcomes (7, 10). Nursing staff in LTCFs must be feel confident in their abilities to care for people with dementia and be competent to provide person-centered dementia care and respond appropriately to BPSD (23). Previous international studies of dementia confidence and care approach have been implemented primarily in developed countries and regions. However, to the best of our knowledge, there is limited published studies in China that explored the same topic. Consequently, we performed the investigation to explore nursing staff confidence to care for people with dementia and their care approach simultaneously as well as demographic factors at LTCFs in mainland China. Accordingly, three research questions were formulated. First, “What is LTCF nursing staff confidence to care for people with dementia?” Second, “what is nursing staff care approach for people with dementia?” Third, “What are the predictors of the confidence to care for people with dementia and dementia care approach among the nursing staff at LTCFs in mainland China?”

## 2. Methods

### 2.1. Designing and setting

A cross-sectional survey of a total of 317 nursing staff at 15 LTCFs in Shandong Province, China, was conducted to explore nursing staff confidence and care approach toward dementia between January and April 2022. Characteristics of the 15 LTCFs are listed in Table 1.

**Table 1:** Characteristics of the 15 long-term care facilities (LTCFs) in 7 cities of Shandong Province, China.

City	Number of LTCF	Nursing staff (*N*)	Ownership		
			Government	Government and private	Private
Jinan	9	228	3	1	5
Qingdao	1	15	0	1	0
Linyi	1	30	0	1	0
Yantai	1	13	1	0	0
Weihai	1	10	0	1	0
Zibo	1	10	1	0	0
Jining	1	11	0	0	1
Total	15	317	5	4	6

### 2.2. Participants and procedure

A convenience sample, of nursing staff working in LTCFs was invited to participate. Inclusion criteria were: the nursing staff (a) had direct care with people with dementia during their daily work for at least 1 month, (b) were able to read, understand, and express verbally in Chinese, and (c) consented to participate in this study. The nursing staff who were currently involved in other research projects were excluded from this study.

Due to the COVID-19 pandemic and LTCF shut-down in China, data were collected both by paper-and-pencil and online methods. Managers of LTCFs were contacted by our investigators (i.e., H.W.C., N.Z., and L.L.) to invite the nursing staff who worked in their facilities to participate. As the recommended minimum sample size was 5–10 times the number of variables in the study (10 variables were included in this study) (24), and given 20% of invalid questionnaires, the final estimated minimum sample size was 120. A sample of 317 was obtained. The sample was sufficient to detect statistically significant results. After explaining the purpose, significance, and requirements of the study in a consistent manner, investigators sent paper questionnaires or the link to the electronic questionnaires (Wenjuanxing-free version) to facility managers. Managers obtained oral informed consent and dispensed questionnaires to the nursing staff. Completed questionnaires were returned to the investigators immediately. The time needed for the nursing staff to complete the questionnaire was approximately 15 to 30 min.

### 2.3. Measurements

#### 2.3.1. Questionnaire

Participants responded to demographic questions including age, gender, educational level, marital status, monthly income, months of caring for people with dementia and experience in dementia-related education.

#### 2.3.2. DKAT 2

The Dementia Knowledge Assessment Tool Version 2 (DKAT 2) was used to assess care workers’ knowledge of dementia. The DKAT 2 contains a total of 21 items. The possible range of scores is 0 to 21 and high total scores indicate greater knowledge of dementia (25). The Chinese version of DKAT 2 has an acceptable internal consistency with a Cronbach’s alpha coefficient of 0.70 (20), which was used to assess the level of dementia knowledge among nursing staff in LTCFs (7). The Cronbach’s alpha coefficient in the current study was 0.68.

#### 2.3.3. CODE

The Confidence in Dementia Scale (CODE) assessed nursing staff confidence to care for people with dementia in general hospital settings. It is an unidimensional questionnaire with nine items (26). The response options are on a 5-point Likert scale with anchored ratings of “not confident (scored 1 point),” “somewhat confident (scored 3 points),” and “very confident (scored 5 points).” The possible total scores ranged from 9 to 45 with the Cronbach’s alpha coefficient ranging from 0.87 to 0.91, supporting its application as a commonly used research instrument. Scerri et al. (10) used the CODE to evaluate the confidence in dementia care among nursing staff in LTCFs. The Chinese version of CODE included three dimensions, i.e., confidence in early-stage dementia (items 1, 2, and 4), confidence in middle or late-stage dementia (items 3, 5, and 6), and confidence in dementia as a whole (items 7 to 9), with the Cronbach’s alpha ranging from 0.824 to 0.897 and it has been already stressed its good psychometric properties (27). In the present study, the content validity index was 0.89 and the Cronbach’s alpha coefficient was 0.87.

#### 2.3.4. ADCQ

The Approach to Advanced Dementia Care Questionnaire (ADCQ) developed by Normann et al. (21) was used to evaluate the nursing staff approach to caring for people with dementia. This questionnaire simulated a background scenario of a woman with severe dementia and typical behaviors of a person with dementia. It contains 13 items divided into five dimensions measuring emphasis on the past or the present, correction of behavior, aim of the communication, orientation of time, place, and situation as well as whether confusion had any meaning for the people with dementia. And each item includes 2 statements, i.e., one reality-oriented dementia care approach (scored 0 point) and another person-centered dementia care approach (scored 1 point). The ADCQ scores ranged from 0 to 13, with higher scores indicating a person-centered approach to dementia care. The Chinese version of the ADCQ of a cross-sectional survey revealed a content validity index of 0.92 and a Cronbach’s alpha coefficient of 0.60, showing high reliability (20). In this study, the Cronbach’s alpha coefficient was 0.602. Permission to use all measurements were obtained.

### 2.4. Statistical analysis

Statistical Product and Service Solutions (SPSS, Version 25.0) software was used for data analysis. Descriptive analysis including the calculation of mean and standard deviations was applied to describe the demographics, CODE, ADCQ scores. We first performed the normality test and homogeneity test of variance. Then, independent *t*-tests and one-way analysis of variance (ANOVA) were used to detect the difference in CODE and ADCQ scores with demographic characteristics. Pearson’s correlation analysis were conducted to determine the association among the DKAT 2, CODE, and ADCQ scores. Multivariate linear regression analysis with “stepwise” selection procedure were used to determine the predictive factors of CODE and ADCQ scores. All statistical tests were two-tailed and statistical significance was set at 0.05.

### 2.5. Ethics

The study was conducted in accordance with the guidelines for research involving humans following the Helsinki Declaration and obtained an exemption from requiring ethics approval from the Ethics Committee of the Affiliated Hospital of Shandong University of Traditional Chinese Medicine (No. 2022-0013). And permissions to use the measurements were obtained from its developers. Participation was voluntary and personal information was kept confidential. A completely anonymous and confidential study was conducted with the data maintained and used exclusively in the current researchers.

## 3. Results

### 3.1. Demographic characteristics of participants

A total of 317 out of the 323 questionnaires administered were received with the high response rate of 98.14%. Nursing staff were aged between 19 and 60 years (mean = 35.61 years, SD = 9.79). Demographic characteristics of the nursing staff were shown in Table 2. The majority of participants female (78.23%). A total of 198 (62.46%) of the staff held a Junior College degree or below, while a total of 160 (50.47%) nursing staff reported that they had experience of more than 3 years in caring for people with dementia.

**Table 2:** Demographic characteristics of participants (*N* = 317).

Variable	Category	Number of nursing staff (%)
Gender	Male	69 (21.77)
	Female	248 (78.23)
Educational level	Technical secondary school or below	64 (20.19)
	Junior college	134 (42.27)
	Bachelor degree or above	119 (37.54)
Marital status	Unmarried	150 (47.32)
	Married	167 (52.68)
Monthly income (RMB)	<3,000	54 (17.04)
	3,000–4,999	185 (58.36)
	5,000–6,999	52 (16.40)
	≥7,000	26 (8.20)
Months of caring for people with dementia	1–36	157 (49.53)
	≥36	160 (50.47)
Experience in dementia-related learning	No	93 (29.34)
	Yes	224 (70.66)

### 3.2. Content domain scores for CODE and ADCQ

The content domain scores for CODE and ADCQ are presented in Table 3. The CODE scores ranged from 11 to 45 with an average of 28.77 (SD = 8.98), presenting a moderate level of confidence to care for people with dementia. The lowest scores were achieved in the dimension of “confidence in middle or late-stage dementia” (mean = 9.27, SD = 3.58). The ADCQ scores ranged from 2 to 12 with the average of 6.21 (SD = 1.96) indicating that nursing staff were likely to adopt the reality-oriented instead of a person-centered approach to care. Higher scores was reported for the “emphasis on the past or the present” dimension (1.80 ± 0.56). The dimension with the lowest score was “whether confusion had any meaning for people with dementia (0.81 ± 0.39), while only 18.93% (*n* = 60) of nursing staff believed that confusion made sense to the simulated woman with severe dementia.

**Table 3:** Content domain scores for CODE and ADCQ (*N* = 317).

Variable	Category	Score (Mean ± SD)	Range
CODE	Confidence in early-stage dementia	9.89 ± 3.32	3–15
	Confidence in dementia as a whole	9.61 ± 3.46	3–15
	Confidence in middle or late-stage dementia	9.27 ± 3.58	3–15
	Total	28.77 ± 8.98	9–45
ADCQ	Emphasis on the past or the present	1.80 ± 0.56	0–3
	Correction of behavior	1.21 ± 0.74	0–3
	Aim of the communication	1.20 ± 0.87	0–3
	Orientation of time, place, and situation	1.18 ± 0.66	0–3
	Whether confusion had any meaning for the people with dementia	0.81 ± 0.39	0–1
	Total	6.21 ± 1.96	0–13

### 3.3. Variations in CODE and ADCQ scores based on demographic characteristics

Significant relationships between demographic characteristics and CODE and ADCQ were noted and are presented in Table 4. The results showed that nursing staff with higher levels of education and more work experience in caring for people with dementia had statistically significant higher confidence scores (*p* < 0.01). Moreover, participants who had dementia-related learning experiences demonstrated greater confidence than those who did not receive dementia-specific education (*p* = 0.046). In addition, statistical analysis of ADCQ score showed that nursing staff who were older, with higher education, married, having more months of caring, and participating in educational programs reported significantly greater patient-cantered care with *p*-values of 0.001, 0.000, 0.002, 0.000, and 0.001, respectively.

**Table 4:** CODE and ADCQ scores based on the demographic characteristics (*N* = 317).

Variable	Category	CODE			ADCQ		
		Mean ± SD	t/r	*p*	Mean ± SD	t/r	*p*
Age (years)	–	28.77 ± 8.98	–0.009	0.875	6.21 ± 1.96	0.184	0.001*
Gender	Male	29.41 ± 8.88	0.661	0.509	6.01 ± 1.79	–0.930	0.353
	Female	28.60 ± 9.02			6.26 ± 1.99		
Educational level	Technical secondary school or below	20.22 ± 7.13	68.195	0.000*	5.55 ± 2.07	10.908	0.000*
	Junior College	28.39 ± 7.56			5.99 ± 1.77		
	Bachelor degree or above	33.81 ± 7.69			6.82 ± 1.94		
Marital Status	Unmarried	28.49 ± 8.98	–0.525	0.600	5.80 ± 1.84	–3.588	0.000*
	Married	29.02 ± 9.00			6.57 ± 1.99		
Monthly income (RMB)	<3000	28.00 ± 8.57	0.329	0.804	5.87 ± 1.60	1.042	0.374
	3000–4999	28.87 ± 9.16			6.33 ± 1.91		
	5000–6999	28.58 ± 9.36			6.02 ± 2.41		
	≥7000	30.08 ± 7.99			6.42 ± 1.94		
Months of caring for people with dementia	1–36	24.80 ± 7.66	–8.679	0.000*	5.71±1.84	–4.667	0.000*
	≥36	32.68 ± 8.47			6.70 ± 1.95		
Experience in dementia-related learning	No	27.22 ± 8.87	–1.999	0.046*	5.62 ± 1.75	–3.490	0.001*
	Yes	29.42 ± 8.97			6.45 ± 1.99		

SD, standard deviation; CODE, Confidence in Dementia Scale; ADCQ, Approach to Advanced Dementia Care Questionnaire. *p < 0.05.

### 3.4. Relationships among the scores of DKAT 2, CODE, and ADCQ

A positive correlation was detected between DKAT 2 and CODE scores (*r* = 0.612, *p* < 0.001) and between CODE and ADCQ scores (*r* = 0.401, *p* < 0.001)indicating significant relationships between dementia knowledge and confidence to care for people with dementia and confidence and person-centered care. However, the DKAT 2 scores were weakly correlated with ADCQ scores (*r* = 0.327, *p* < 0.001; Table 5).

**Table 5:** Relationships among the scores of DKAT 2, CODE, and ADCQ (*N* = 317).

Variable	DKAT 2	CODE	ADCQ
DKAT 2	1		
CODE	0.612 (<0.001)	1	
ADCQ	0.327 (<0.001)	0.401 (<0.001)	1

### 3.5. Predictors for the CODE and ADCQ scores

The results of the multiple linear regression analysis are presented in Table 6. The analysis included variables significantly associated with the CODE and ADCQ scores determined by the above univariate and correlation analyses. The results showed that education level, months of caring for people with dementia, and dementia knowledge were independent predictors of CODE (*F* = 84.05, *p* = 0.000), and explained 51.2% of the total variance in CODE scores. Moreover, four factors, i.e., age, dementia-related education, dementia knowledge, and confidence in dementia were associated with the care approach (*F* = 14.412, *p* = 0.000), accounting for 22.9% of the total variance in ADCQ scores.

**Table 6:** Multiple linear regression analysis of the CODE and ADCQ scores (*N* = 317).

Independent variable		B	SE	**β**	t	*p*
CODE	(Constant)	–2.914	2.159		–1.350	0.178
	Educational level (technical secondary school and below = 1)	3.769	0.533	0.311	7.066	0.000*
	Months of caring for people with dementia (1–36 = 1)	4.179	0.790	0.233	5.293	0.000*
	Experience in dementia-related learning (yes = 1)	–1.306	0.817	–0.066	–1.598	0.111
	Dementia knowledge (DKAT 2 scores)	1.302	0.140	0.415	9.309	0.000*
	*R* = 0.720, *R*^2^ = 0.519, Adjusted *R*^2^ = 0.512, *F* = 84.05 (*p* = 0.000)					
ADCQ	(Constant)	0.214	0.685		0.312	0.755
	Age (years)	0.026	0.012	0.132	2.194	0.029*
	Educational level (technical secondary school and below = 1)	–0.005	0.158	–0.002	–0.029	0.977
	Marital status (unmarried = 1)	0.463	0.241	0.118	1.926	0.055
	Months of caring for people with dementia (1–36 = 1)	–0.164	0.242	–0.042	–0.680	0.497
	Experience in dementia-related learning (yes = 1)	0.482	0.224	0.114	2.156	0.032*
	Dementia knowledge (DKAT 2 scores)	0.134	0.042	0.199	3.183	0.002*
	Confidence in dementia (CODE scores)	0.063	0.016	0.291	4.083	0.000*
	*R* = 0.496, *R*^2^ = 0.246, Adjusted *R*^2^ = 0.229, *F* = 14.412 (*p* = 0.000)					

DKAT 2, Dementia Knowledge Assessment Tool Version 2; CODE, Confidence in Dementia Scale; ADCQ, Approach to advanced dementia care questionnaire. *p < 0.05.

## 4. Discussion

This study was novel in that it is the first report in mainland China to examine nursing staff confidence in dementia care and care approach for people with dementia in LTCFs. It is critically important to understand the perspectives of LTCF nursing staff regarding dementia care in order to facilitate the development of continuing educational interventions and training programs to boost the quality of dementia care. The present study revealed that LTCF nursing staff did not have the adequate confidence in dementia care and a person-centered approach to dementia care.

The results of our study indicated that the nursing staff confidence in caring people with dementia and dealing with dementia-related situations were generally at moderate level. Compared to the previous studies, the CODE scores reported in this study (28.77 ± 8.98) were lower than those of the nursing staff working at the public nursing and residential homes in Malta (32.18 ± 6.52) using the same assessment tool (11). Given that knowledge was positively correlated with confidence, these results were probably due to deficient knowledge among nursing staff. Additionally, lower education correlated with confidence suggesting that the nursing staff with lower educational level showed lower confidence in dementia care. A questionnaire survey conducted in Australia reported that 56% LTCFs nursing staff had 2 to 3 years of formal higher education (28). In this study, 62.46% nursing staff had educational levels of junior college or below. Furthermore, there is a consensus that fewer number of years on the job and the limited dementia-related training opportunities correlated with nursing staff confidence in caring for people with dementia (6). The findings of this current study were consistent with this consensus. These results could be explained by the facts that nursing staff with more education and exposure and closer contact with people with dementia would have a more intuitive and profound understanding of dementia (29). Moreover, our results showed that the scores of “confidence in middle or late-stage dementia” were lower than those of the other two dimensions (i.e., “confidence in early-stage dementia” and “confidence in dementia as a whole”), suggesting that nursing staff had low confidence in caring for the people with dementia at middle and late stage. One possible explanation was that nursing staff lacked knowledge and skills to care for people with dementia in later stages. Therefore, nursing staff should be offered more in-depth comprehensive dementia-specific training, such as recognition of and how to respond to BPSDs exhibited at middle or late-stages. This will enhance staff understanding of dementia and promote their confidence in dementia care (30).

As the gold standard of dementia care (31), person-centered care plays a positive role in preventing and responding to behavioral and psychological symptoms of dementia, and provision of quality of life, while relieving the stress and burden of nursing staff (18, 32). However, as revealed in our study, not all nursing staff employed a person-centered care approach for people with dementia. These results were consistent with those reported in the previous studies (7, 20). These results could be explained as follows. First, nearly one-third (29.34%) of the nursing staff in this study never received any dementia-related education, leading to a knowledge blind spot about the person-centered care approach. Similarly, a previous study showed 32.9% of nursing staff in the LTCFs were never trained in any dementia care program (7). Second, nursing staff with dementia-related education showed better understanding and higher likelihood to implement a person-centered care approach compared to those without education. Our results showed that only a few nursing staff who thought “confusion was meaningful” for people with dementia considered the person-centered care approach as the most rewarding practice. It was encouraging to see that person-centered care education was an effective approach to prepare nursing staff with specialized knowledge and skills, as reported by Zhao et al. (33). Third, interestingly, our results showed that age was associated with nursing staff care approach, which was rarely reported in previous literature. It was reasonably speculated that older nursing staff were more experienced in caring for people with dementia and would focus more on personality instead of custodial care (34). Last, the results of the multiple linear regression analysis showed that dementia knowledge and confidence in dementia care among LTCF nursing staff were important factors contributing to the selection of person-centered care approach. This means that improving nursing staff knowledge and confidence in dementia care could improve their use of person-centered care. Therefore, efficient delivery and dissemination of educational training which have already been shown to improve the knowledge as well as the confidence in dementia care were considered important ways of motivating the nursing staff in LTCFs to implement the person-centered care approach (35).

Study results correspond with the Holton’s model. Higher level of knowledge in staff members is correlated with higher confidence in dementia care and person-centered care approach toward nursing staff. Additionally, study findings emphasize the key strategy to enable nursing staff in LTCFs to provide high-quality care for people with dementia is continuing education which will improve their understanding of complex dementia management (36). From the view of LTCFs managers, more opportunities of continuing education programs should be provided to help nursing staff acquire dementia knowledge and improve their related confidence and learn person-centered care approaches. Virtual dementia education was an effective intervention for short-term and professional nursing staff to enhance their knowledge and care skills (37). Further, peer support discussion groups and e-learning with mentoring support could be offered to enhance the care workers’ knowledge, attitude, and competence (38, 39). These interventions could also be designed and investigated for feasibility among nursing staff in the LTCFs under the social and cultural environment in China. From the government’s perspective, policies and funding are urgently needed to address a qualified workforce prepared to respond to increased numbers of people with dementia in the LTCFs. Research should be undertaken to tailor interventions to increase the knowledge level among nursing staff, and enhance their confidence and care approach in their future careers.

## 5. Limitations of the study and future recommendations

Several limitations in this study were noted and should be addressed in the future studies. First, the study was conducted using a convenience sample and the caregiver populations in the 7 districts of Shandong Province may not represent adequately the LTCFs of other geographic locations of China, limiting generalization of the findings. Second, the present study was a cross-sectional survey with the multivariate regression models developed for statistical purposes rather than theoretical objectives. The current study utilized paper-and-pencil and online methods to collect data on nursing staff confidence and care approach toward dementia in LTCFs. The mixing of data collection methods potentially might introduce bias. Future studies are necessary to further verify findings.

## 6. Conclusion

This present study revealed that Chinese nursing staff in the LTCFs were not equipped with sufficient confidence in dementia care and tended not to employ a person-centered care approach for people with dementia. Our results showed that among the nursing staff, the higher confidence in dementia was associated with higher educational level, more months of caring dementia patients, and relatively richer knowledge of dementia, whereas age, experience in dementia-learning, and knowledge and confidence in dementia care were independent predictors of nursing staff care approach. These results highlighted the necessity of continued education strategies for nursing staff in order to meet the growing demand for dementia care services in the LTCFs. Future studies should focus on enhancing the specialized dementia care educational interventions for nursing staff so as to equip them with dementia knowledge, boost their confidence, and identify their selection of person-centered care under the social and cultural environment in China.

## Data availability statement

The datasets presented in this article are not readily available because the first author of this study is a graduate student in school, and the data involved in this study is also used to apply for master’s degree thesis. Relevant academic achievements have not been published, and the copyright belongs to the original author, so it cannot be publicly provided for the time being. Requests to access the datasets should be directed to HC, haiwenchen1207@163.com.

## Ethics statement

The studies involving humans were approved by the Ethics Committee of the Affiliated Hospital of Shandong University of Traditional Chinese Medicine. The studies were conducted in accordance with the local legislation and institutional requirements. The participants provided their written informed consent to participate in this study.

## Author contributions

HC was the Principal Investigator and responsible for data collection, statistical analysis, and drafting the manuscript. JS reviewed and revised the manuscript, including the results section. NZ and NL had responsibility for the formal analysis and reviewing the manuscript. QJ contributed to methodology and validation. XL, LL, and YL contributed to data collection and data curation. All authors contributed to the article and approved the submitted version.

## Funding

Open access funding provided by Shandong University of Traditional Chinese Medicine.

## Conflict of interest

The authors declare that the research was conducted in the absence of any commercial or financial relationships that could be construed as a potential conflict of interest.

## Publisher’s note

All claims expressed in this article are solely those of the authors and do not necessarily represent those of their affiliated organizations, or those of the publisher, the editors and the reviewers. Any product that may be evaluated in this article, or claim that may be made by its manufacturer, is not guaranteed or endorsed by the publisher.
